# Molecular characterization of *mcr-1.1*-harboring multidrug-resistant *Escherichia coli* isolates from chicken in the United Arab Emirates: implications for one health surveillance

**DOI:** 10.3389/fvets.2025.1714397

**Published:** 2026-01-20

**Authors:** Hazim O. Khalifa, Mohammed Elbediwi, Temesgen Mohammed, Afra Abdalla, Mohamed-Yousif Ibrahim Mohamed, Glindya Bhagya Lakshmi, Ihab Habib

**Affiliations:** 1 Department of Veterinary Medicine, College of Agriculture and Veterinary Medicine, United Arab Emirates University, Al-Ain, United Arab Emirates; 2 Institute for Medical Microbiology and Virology, Carl von Ossietzky University Oldenburg, Oldenburg, Germany; 3 Animal Health Research Institute, Agriculture Research Centre, Cairo, Egypt; 4 ASPIRE Research Institute for Food Security in the Drylands (ARIFSID), United Arab Emirates University, Al Ain, United Arab Emirates

**Keywords:** antimicrobial resistance, chicken, colistin resistance, mcr-1.1, one health

## Abstract

**Background:**

The *mcr-1.1* gene, conferring resistance to colistin, is a significant threat to public health, particularly due to its capacity for horizontal gene transfer between diverse bacterial populations in humans, animals, and the food chain. This study investigated the occurrence, phenotypic antimicrobial resistance (AMR) profiles, genetic characteristics, and plasmid characterization of *mcr-1.1*-producing *Escherichia coli* isolates from different samples in the United Arab Emirates (UAE).

**Methods:**

A total of 333 Gram-negative isolates were screened by PCR for the detection of *mcr* genes. Antimicrobial susceptibility testing, whole genome sequencing (WGS), plasmid analysis, and Phylogenomic typing were performed to assess AMR determinants, plasmid replicons, genetic contexts of *mcr-1.1*, and genetic relatedness between isolates from the UAE and neighboring countries.

**Results:**

We identified 15 *mcr-1.1*-positive *E. coli* strains, all from chicken cecal samples. These isolates exhibited multidrug resistance (MDR) to various classes of antibiotics, including *β*-lactams, tetracyclines, quinolones, and aminoglycosides. WGS of 15 *mcr*-positive *E. coli* isolates revealed the presence of multiple AMR genes along with mutations in quinolone resistance genes (*gyrA*, *parC*). Plasmid analysis revealed that all *mcr-1.1*-positive strains carried at least one plasmid replicon, with the IncF and IncI plasmids being the most prevalent. Notably, the *mcr-1.1* gene was located on IncI2 and IncX4 plasmids, with comparative analysis showing high sequence homology to plasmids from *E. coli* strains originating from humans and animals in multiple countries. The plasmids’ high sequence homology across diverse geographical regions provides genomic evidence consistent with possible cross-border dissemination of *mcr-1.1*, facilitating the spread of colistin resistance. Genetic mapping of the *mcr-1.1* gene revealed distinct genetic contexts depending on the plasmid type, with genes such as *nikA*, *nikB*, and *pap2* flanking the gene on IncI2 and IncX4 plasmids. Clonal analysis using whole-genome sequencing identified 12 different sequence types (STs) among the 15 isolates, with ST10, ST117, and ST162 being the most prevalent. Core genome multilocus sequence typing demonstrated genetic relatedness between isolates from the United Arab Emirates (UAE) and neighboring countries, indicating potential transmission across borders via the food chain.

**Conclusion:**

Our findings highlight the complex interaction between plasmid-mediated colistin resistance, AMR, and virulence traits in *E. coli* from the food chain. The genetic and plasmid similarities between *mcr-1.1-*producing isolates across multiple countries emphasize the risk of possible dissemination and the potential risk of cross-border dissemination through globally traded food products. This study underscores the need for regional and global surveillance and control measures to mitigate the spread of this multidrug-resistant pathogen.

## 1 Introduction

Antimicrobial resistance (AMR) is a growing global health crisis that occurs when bacteria and fungi develop resistance to antimicrobial agents, posing a significant threat to modern medicine, agriculture, and food security (1, 2). Unlike sudden health emergencies like viral pandemics, AMR has developed gradually since the introduction of the first antibiotics and was recognized by the World Health Organization (WHO) in 2019 as one of the top 10 global public health threats (3). Antimicrobial medicines are the cornerstone of healthcare, enabling the treatment of common infections and the success of life-saving procedures such as cancer chemotherapy, organ transplantation, and major surgeries (4). However, the rise of drug-resistant pathogens jeopardizes these advancements and extends its impact to animal and plant health, reducing farm productivity and threatening food security (5). The issue is further exacerbated by the misuse and overuse of antibiotics in clinical settings, agriculture, animal healthcare, and even during crises like wars (2–5). Often referred to as the “Silent Pandemic,” AMR demands immediate intervention. Without effective measures, it is projected that by 2050, AMR could become the leading cause of mortality worldwide, with annual deaths potentially reaching 10 million, far surpassing current figures of over 1.2 million deaths annually attributed to drug-resistant infections (5). This escalating threat not only imposes a significant burden on healthcare systems through increased costs and prolonged hospital stays but also undermines economic productivity, underscoring the urgent need for global action to combat AMR.

Colistin, a member of the polymyxin antibiotic family, combats Gram-negative bacteria by disrupting their outer membranes (5–8). However, its increased usage has contributed to a rising prevalence of colistin resistance globally (9), with rates exceeding the worldwide average of 10% in low- and middle-income regions, particularly from the Mediterranean to Southeast Asia (10). The primary mechanism of colistin resistance in Enterobacterales involves structural alterations of the lipid A component of lipopolysaccharides (LPS) through cationic substitutions (11). The incorporation of cationic molecules like phosphoethanolamine (PEtN) or 4-amino-l-arabinose reduces the negative charge of LPS, diminishing the negative charge on the bacterial surface (12). This modification decreases colistin’s binding affinity for the bacterial outer membrane. Initially, resistance was attributed to chromosomal mutations in two-component regulatory systems such as phoP/Q, pmrA/B, crrA/B, and the regulator mgrB (13). Additionally, the discovery of the plasmid-borne colistin resistance gene *mcr-1* in 2015 marked a significant development (7, 8, 14). This gene encodes a phosphoethanolamine transferase enzyme that modifies lipid A by adding PEtN (15). The *mcr-1* gene was first identified in *Escherichia coli* from a swine farm in China. Since its discovery, over 10 *mcr* variants have been detected in 27 bacterial species across all six continents, underscoring the rapid global dissemination of colistin resistance (10).

Monitoring *mcr*-producing bacteria across animals, humans, and the food chain is essential for identifying potential public health risks, guiding responsible antimicrobial use, and mitigating the spread of AMR within the One Health framework. These bacteria, including zoonotic pathogens such as *E. coli*, *Salmonella Typhimurium*, and *Klebsiella pneumoniae*, have been found to harbor *mcr* genes in various reservoirs, including animal intestines, human carriers, and food products (7, 8, 14, 16). Their detection is particularly critical as they can be transmitted to humans through direct animal contact, consumption of contaminated or undercooked food, or cross-contamination during food preparation (7, 8). *mcr* producers have demonstrated the ability to disseminate both locally and globally (17), often co-carrying additional AMR genes, and are frequently associated with multidrug resistance, posing a serious threat to public health. Furthermore, because the poultry cecum harbors dense Enterobacterales communities historically exposed to on-farm polymyxin use, it functions as a hotspot for horizontal transfer and persistence of plasmid-borne mcr genes—making cecal sampling at slaughter a sensitive reservoir indicator within One Health surveillance (7). Despite increasing global concern, limited information is available regarding the prevalence and genetic characteristics of *mcr*-producing Gram-negative bacteria across these interconnected sectors in the UAE. To address this knowledge gap, the current study aims to assess the occurrence, phenotypic traits, and genetic resistance profiles of *mcr*-producing Gram-negative bacteria isolated from different sources in the UAE. Additionally, it seeks to investigate the genetic relationships between *mcr*-producing *E. coli* from these different sources.

## 2 Materials and methods

### 2.1 Bacterial isolates

In this study, 333 non-duplicate Gram-negative bacterial isolates (one bacterial species for every sample) were screened for the *mcr* gene using PCR (Supplementary Figure S1). The isolates included 148 from chickens, 114 from human clinical specimens, and 71 from animals. All the samples were initially cultured on MacConkey agar supplemented with cefotaxime 4 μg/mL. From each sample, distinct colonies showing different morphology or species identity (based on preliminary biochemical testing or MALDI-TOF identification) were subcultured and preserved. Only one isolate per bacterial species per sample was included to avoid duplication. Isolates were selected independently of their phenotypic resistance pattern, ensuring an unbiased representation of Gram-negative species present in each sample. Chicken isolates were collected from 77 samples (59 cecal droppings and 18 cecal contents) obtained over 11 months (June 2023–April 2024) from farmhouses and slaughterhouses of two major poultry companies in Al Ain, UAE. Sampling covered 18 poultry houses across six farms from the first company and nine houses across three farms from the second. Human isolates were obtained from rectal swabs of pregnant women between October 2024 and September 2025, while animal isolates were collected in 2024 from rectal, fecal, water, and feed swabs of various aquatic animals. Human sample collection was approved by the Abu Dhabi Health Research and Technology Ethics Committee (DOH/ADHRTC/2024/1487, 17th May, 2024), while animal sample collection was approved by the UAEU Animal Ethics Committee (ERA_2025_6066). The detection was performed using the primer pair MCR-1-F2 (5′-CTCATGATGCAGCATACTTC-3′) and MCR-1-R2 (5′-CGAATGGAGTGTGCGGTG-3′), as previously described in our earlier work (14). In this study, we identified 15 *mcr*-positive *Escherichia coli* isolates using PCR, which were subsequently characterized both phenotypically and genotypically to determine their resistance patterns and to perform phylogenomic analysis.

### 2.2 Antimicrobial sensitivity testing

Antimicrobial susceptibility testing was performed on all *mcr-1.1*-harboring isolates according to the protocols outlined by the Clinical and Laboratory Standards Institute (CLSI, 2022) (18). The disk diffusion method (Kirby–Bauer technique) was utilized for this purpose, and *E. coli* ATCC 25922 was included as the reference quality control strain. The tested antibiotics encompassed several major classes, including *β*-lactams—ampicillin (AMP, 10 μg), amoxicillin–clavulanic acid (AMC, 20/10 μg), cefoperazone (CFP, 75 μg), ceftriaxone (CRO, 30 μg), cefoxitin (FOX, 30 μg), meropenem (MEM, 10 μg), and imipenem (IPM, 10 μg); quinolones—ciprofloxacin (CIP, 5 μg) and nalidixic acid (NAL, 30 μg); aminoglycosides—gentamicin (GEN, 10 μg) and amikacin (AMK, 30 μg); along with tetracycline (TET, 30 μg) and chloramphenicol (CHL, 30 μg). Colistin resistance was determined separately using the broth microdilution method to establish minimum inhibitory concentrations (MICs), following CLSI guidelines published in 2022 (18).

### 2.3 Phenotypic detection of extended-spectrum *β*-lactamase and carbapenemases production

Phenotypic confirmation of extended-spectrum β-lactamase (ESBL) production was carried out using the CLSI-recommended confirmatory double-disc synergy test (DDST). This method involved placing ceftriaxone (CTA; 30 μg) and ceftazidime (CAZ; 30 μg) discs individually, as well as in combination with clavulanic acid (CA; 10 μg) (MiMedia, Maharashtra, India). A result was considered positive if the inhibition zone around the CTA or CAZ disc combined with clavulanic acid was at least 5 mm larger than that of the corresponding antibiotic disc alone (18). Additionally, carbapenemase production was assessed using the modified carbapenem inactivation method (mCIM), as described in our previous publications (19, 20).

### 2.4 Whole genome sequence (WGS) and bioinformatics analysis

WGS was performed on all *mcr-1.1*-producing isolates identified in this study to validate phenotypic findings and provide deeper insights into the underlying molecular resistance mechanisms. Genomic DNA was extracted using the Wizard^®^ Genomic DNA Purification Kit (Promega, Madison, WI, USA) according to the manufacturer’s instructions. The concentration and purity of the DNA were assessed using both the ScanDrop^2^ Nano-volume spectrophotometer (Analytikjena, Life Science, Germany) and the NanoDrop 1,000 UV–Vis spectrophotometer (Thermo Scientific, Waltham, MA, USA). Sequencing was outsourced to Novogene and conducted on the Illumina NovaSeq platform (paired-end 150 bp reads).

Bioinformatics analysis of the WGS data involved several steps. Raw reads were quality-checked and *de novo* assembled using SPAdes v4.0.1 with the “careful correction” option enabled to minimize mismatches, and k-mer sizes were automatically selected by the software (21). Identification of antimicrobial resistance genes, virulence factors, and plasmid replicon types was carried out using the Abricate tool.^1^ The databases used were ResFinder v4.1 (accessed 2025-07-01), PlasmidFinder v2.1 (accessed 2025-07-01), and VirulenceFinder v2.0 (accessed 2025-07-01) (22). For plasmid reconstruction, reads were assembled using Unicycler (23), and the resulting GFA files were analysed with gplas 2 (24) for plasmid component classification. It is important to note that, due to the short-read sequencing technology, the IncI2 and IncX4 plasmids discussed in this study are inferred from contigs and represent high-confidence draft assemblies, not complete circular molecules. Reconstructed plasmid contigs were further aligned against the NCBI nucleotide BLAST database for homology analysis. Moreover, we used (≥90% identity, ≥60% coverage) thresholds for gene and plasmid identification. The genetic environment surrounding *mcr-1.1* genes was visualized using Easyfig 2 (22).

To investigate phylogenomic relationships, core genome multilocus sequence typing (cgMLST) analysis was performed using cgMLSTFinder v1.2 (25, 26) and the *E. coli* cgMLST v1.0 scheme (2,513 loci). Isolates were considered closely related if they shared fewer than 10 allele differences. The resulting tree was visualized using R packages including ggtree (10.18129/B9.bioc.ggtree), ape (10.32614/CRAN.package.ape), dplyr,^2^ tidyr,^3^ and ggplot2.^4^ The R codes used for tree visualization have been deposited in a publicly accessible GitHub repository.^5^ This analysis included the 15 *mcr-1.1*-positive *E. coli* isolates from the current study alongside 80 additional *mcr-1.1*-positive *E. coli* genomes retrieved from the NCBI database, comprising isolates from the United Arab Emirates (*n* = 57), Qatar (*n* = 21), and Saudi Arabia (*n* = 2), collected between 2017 and 2024 (Supplementary Table S1). These isolates originated from diverse sources: humans (*n* = 47), chicken meat (*n* = 26), live poultry (*n* = 5), and other live animals (*n* = 2).

## 3 Results

### 3.1 Identification and phenotypic characterization of the *mcr*-positive isolates

In this study, the *mcr* gene was detected in 15 isolates originating from chicken cecal samples. No *mcr*-positive isolates were identified from other sources, including human clinical specimens or animal samples. All *mcr*-positive isolates were subsequently characterized both phenotypically and genotypcally to elucidate their resistance mechanisms. Phenotypically, multidrug resistance and ESBL production were confirmed in all isolates, based on resistance to at least three antibiotic classes and positive results from the double-disc synergy test, respectively (Table 1). Additionally, all isolates were susceptible to imipenem and meropenem, consistent with the mCIM results, which confirmed that none of the isolates produced carbapenemases. Interestingly, all isolates were susceptible to AMP combined with clavulanic acid, yet resistant to AMP alone, indicating effective inhibition of *β*-lactamases. Regarding other β-lactam antibiotics, 100% of isolates were resistant to CRO and CFP, while 93.3% (14/15) were resistant to FOX. High levels of resistance were also observed for tetracycline TET at 93.3% (14/15), and for the quinolones CIP and NAL, both at 86.7% (13/15). In contrast, moderate resistance was detected against CHL at 60% (9/15), and resistance to aminoglycosides varied from moderate to low, ranging from 60% (9/15) for GEN to 6.7% (1/15) for amikacin AMK.

**Table 1:** Antimicrobial resistance profile of 15 *mcr-1*-producing *E. coli* isolates tested in this study.

No	Isolate ID	Colistin MIC	Resistance phenotype	MDR profile	ESBL production	Carbapenemases production
1	S48C	8	AMP, CRO, TET, FOX, CFP, CHL, NAL, GEN, CIP	Positive	Positive	Negative
2	S64C1	8	AMP, CRO, TET, FOX, CFP, CHL	Positive	Positive	Negative
3	S99C	16	AMP, CRO, TET, FOX, CFP, CHL, NAL, GEN, CIP	Positive	Positive	Negative
4	S115C	8	AMP, CRO, TET, FOX, CFP, CHL, NAL, GEN, CIP	Positive	Positive	Negative
5	S131C	8	AMP, CRO, TET, FOX, CFP, CHL, NAL, GEN, CIP	Positive	Positive	Negative
6	S136C2	8	AMP, CRO, TET, FOX, CFP, CHL, NAL, GEN, CIP	Positive	Positive	Negative
7	S165C	8	AMP, CRO, TET, FOX, CFP, NAL, GEN, CIP	Positive	Positive	Negative
8	S166C1	8	AMP, CRO, TET, CFP, CHL, NAL, GEN, CIP	Positive	Positive	Negative
9	S166C2	8	AMP, CRO, TET, FOX, CFP, NAL, AMK, CIP	Positive	Positive	Negative
10	S167C1	8	AMP, CRO, TET, FOX, CFP	Positive	Positive	Negative
11	S167C2	4	AMP, CRO, TET, FOX, CFP, CHL, NAL, CIP	Positive	Positive	Negative
12	S200C1	4	AMP, CRO, TET, FOX, CFP, CHL, NAL, GEN, CIP	Positive	Positive	Negative
13	S200C2	8	AMP, CRO, TET, FOX, CFP, NAL, GEN, CIP	Positive	Positive	Negative
14	S261C1	8	AMP, CRO, FOX, CFP, NAL, CIP	Positive	Positive	Negative
15	S296C	4	AMP, CRO, TET, FOX, CFP, NAL, CIP	Positive	Positive	Negative

Ampicillin, AMP; ceftriaxone, CRO; Cefoxitin, FOX; Cefoperazone, CFP; Chloramphenicol, CHL; Nalidixic Acid, NAL; gentamicin, GEN; amikacin, AMK; ciprofloxacin, CIP.

### 3.2 Antimicrobial resistance genes and mutations associated with resistance

WGS was performed to comprehensively characterize 15 selected *mcr-1.1*-producing *E. coli* isolates. The full assembly’s quality metrics of the sequenced isolates are provided in Supplementary Table S2. Analysis of *β*-lactamase-encoding genes revealed that members of the *bla*_CTX-M-1_ group were present in 13 isolates. Among these, *bla*_CTX-M-55_ was the most prevalent variant, detected in seven isolates, followed by *bla*_CTX-M-15_ in five isolates and *bla*_CTX-M-8_ in a single isolate (Table 2 and Supplementary Table S3). Extended-spectrum *β*-lactamase (ESBL) variants of *bla*_TEM_ were identified in five isolates: *bla*_TEM-214_ in three isolates, and *bla*_TEM-30_ and *bla*_TEM-21*5*_ each in one isolate. Additionally, non-ESBL *bla*_TEM_ genes were detected in seven isolates, with *bla*_TEM-1B_ and *bla*_TEM-176_ each present in three isolates, and *bla*_TEM-1A_ detected in one isolate. The ESBL gene *bla*_SHV-12_ was identified in a single isolate. In addition to β-lactam resistance genes, the isolates carried a diverse array of AMR determinants (Table 2 and Supplementary Table S3). Aminoglycoside resistance genes were detected in all isolates, with *aph(3′)-Ia*, *aph(3″)-Ib*, *aadA2*, and *aph(6)-Id* each present in 10 isolates. Quinolone resistance genes were found in seven isolates, with *qnrS1* being the most common (six isolates). Trimethoprim resistance genes were identified in nine isolates, with *dfrA14* being the most frequent variant (five isolates). Sulfonamide resistance genes were detected in 13 isolates, with *sul3* present in 12 isolates. Tetracycline resistance genes were also identified in 13 isolates, with *tet(A)* consistently detected in all of them. Resistance to phenicols was observed in 12 isolates, with *cmlA1* and *floR* identified in 11 and nine isolates, respectively. Macrolide resistance genes were present in five isolates: three carried both *mph(A)* and *erm(B)*, one carried only *mph(A)*, and one carried only *erm(B)*. Fosfomycin resistance genes were found in five isolates, with *fosA3* detected in four isolates and *fosA4* in one isolate. The lincosamide resistance gene *lnu(F)* was detected in a single isolate. Lastly, disinfectant resistance genes were found in 10 isolates, with *sitABCD* being the most prevalent, identified in nine isolates.

**Table 2:** Full genetic characterization of the 15 *mcr-1*-producing *E. coli* isolates based on whole genome sequencing.

No	Isolate ID	Sequence type (ST)	Resistant genes	Mutations	Virulence genes	Plasmids
1	S48C	ST189	*bla*_CTX-M-55_, *bla*_TEM-214_, *mcr-1.1*, *aac(3)-Iid*, *aadA2*, *aadA22*, *aph(3″)-Ib*, *aph(6)-Id*, *dfrA12*, *catA1*, *erm(B)*, *floR*, *fosA3*, *mph(A)*, *qacE*, *tet(A)*, *tet(M)*, *sul1*, *sul2*, *sul3*	*gyrA* (D87N), *gyrA* (S83L), *parC* (S80I)	*cif, espJ, fimE, fyuA, irp1, irp2, nleB2, papB, yagW/ecpD, ybtA, ybtE, ybtP, ybtQ, ybtT, ybtU*	Col(BS512), IncHI2, IncHI2A, IncY
2	S64C1	ST46	*bla*_CTX-M-15_, *bla*_TEM-30_, *mcr-1.1*, *aac(3)-Iid*, *ant(3″)-Ia*, *aph(3′)-Ia*, *aadA17*, *dfrA12*, *floR*, *cmlA1*, *qnrS1*, *tet(A)*, *sul2*, *sul3*			IncI2(Delta), IncX1, p0111
3	S99C	ST117	*bla*_CTX-M-55_, *mcr-1.1*, *aac(3)-Iid*, *aph(3″)-Ib*, *aph(6)-Id*, *aadA1*, *aadA2*, *cmlA1*, *floR*, *qnrS1*, *sitABCD*, *tet(A)*, *sul2*, *sul3*	*parC* (S80R)	*fimE, yagW/ecpD, yagZ/ecpA*	ColpVC, IncFIB(AP001918), IncFII, IncI2(Delta)
4	S115C	ST359	*bla*_CTX-M-55_, *bla*_TEM-214_, *mcr-1.1*, *aph(3″)-Ib*, *aph(3′)-Ia*, *aph(6)-Id*, *aadA1*, *aadA2*, *catA1*, *cmlA1*, *floR*, *sitABCD*, *tet(A)*, *sul2*, *sul3*	*gyrA* (D87Y), *gyrA* (S83L), *parC* (S80I)	*astA, chuU, fimI, iroC, iroE, iroN, papB, pic, vat, ykgK/ecpR*	IncB/O/K/Z, IncFIB(AP001918), IncFII, IncFII(pHN7A8), IncI1-I(Alpha), IncI2(Delta),p0111
5	S131C	ST117	*bla*_CTX-M-55_, *mcr-1.1*, *aac(3)-Iid*, *aph(3″)-Ib*, *aph(6)-Id*, *aadA1*, *aadA2*, *cmlA1*, *floR*, *qnrS1*, *sitABCD*, *tet(A)*, *sul2*	*parC* (S80R)	*iroC, iroE, iroN, yagZ/ecpA*	ColpVC, IncFIB(AP001918), IncFII, IncI2(Delta)
6	S136C2	ST457	*bla*_SHV-12_, *bla*_TEM-1B_, *mcr-1.1*, *aac(3)-Iid*, *aph(3″)-Ib*, *aph(3′)-Ia*, *aph(6)-Id*, *aadA1*, *aadA2*, *dfrA17*, *cmlA1*, *floR*, *qacE*, *sitABCD*, *tet(A)*, *sul2*, *sul3*	*parC* (S80I), *parE* (S458A)	*astA, chuU, fimI, iroC, iroE, iroN, papB, pic, vat, ykgK/ecpR*	IncFIB(AP001918), IncX4
7	S165C	ST162	*bla*_CTX-M-15_ *, bla*_TEM-1B_ *, mcr-1.1, aac(3)-Iia, aadA2, aadA24, aph(3″)-Ib, aph(3′)-Ia, aph(6)-Id, dfrA14, cmlA1, erm(B), mph(A), sitABCD, tet(A), sul3*	*parC* (S80I), *gyrA* (S83L), *gyrA* (D87N)	*astA, chuU, chuV, entC, fimC, fimD, fimI, papB, yagZ/ecpA, ykgK/ecpR*	IncFIB(AP001918), IncI2(Delta), IncY
8	S166C1	ST101	*bla*_CTX-M-8_, *bla*_TEM-1A_, *mcr-1.1*, *aac(3)-Iia*, *aadA1*, *aph(3″)-Ib*, *aph(3′)-Ia*, *aph(6)-Id*, *dfrA14*, *catA1*, *cmlA1*, *floR*, *mph(A)*, sitABCD*, tet(A)*, *sul2*, *sul3*	*gyrA* (D87Y), *gyrA* (S83L), *parC* (S80I)	*fimE, fimI, yagZ/ecpA*	IncFIB(AP001918), IncFIB(pLF82-PhagePlasmid), IncHI2, IncI1-I(Alpha)
9	S166C2	ST354	*bla*_CTX-M-55_, *bla*_TEM-215_, *mcr-1.1*, *aadA1*, *dfrA14*, *fosA3*, *qnrS1*, *tet(A)*	*parC* (E84G), *parC* (S80I)	*iroC, iroE, iroN, papB, yagZ/ecpA,*	IncFII(pHN7A8), IncI(Gamma), IncI2(Delta)
10	S167C1	ST10	*bla*_CTX-M-55_, *bla*_TEM-214_, *mcr-1.1*, *aac(3)-IV*, *aadA22*, *aph(4)-Ia*, *dfrA14*, *fosA3*, *lnu(F)*, *qnrS1*, *tet(A)*		*astA, chuU, chuV, fes, ykgK/ecpR*	IncFIB(AP001918), IncFII(pHN7A8), IncI1-I(Alpha), IncI2(Delta), p0111
11	S167C2	ST2172	*bla*_CTX-M-15_, *bla*_TEM-176_, *mcr-1.1*, *aadA2*, *ant(3″)-Ia*, *aph(3′)-Ia*, *cmlA1*, *floR*, *sitABCD*, *tet(A)*, *sul3*	*gyrA* (D87N), *gyrA* (S83L), *parC* (S80I)	*fimE, fyuA, iroC, iroE, iroN, irp1, irp2, yagZ/ecpA, ybtA, ybtE, ybtP, ybtQ, ybtS, ybtT, ybtU, ybtX*	IncB/O/K/Z, IncFIA, IncFIB(AP001918), IncI1-I(Alpha), IncI2(Delta), IncX1, p0111
12	S200C1	ST1771	*bla*_CTX-M-15_, *mcr-1.1*, *aac(3)-Iia*, *aph(3″)-Ib*, *aph(3′)-Ia*, *aph(6)-Id*, *aadA2*, *aadA24*, *dfrA1*, *cmlA1*, *erm(B), floR*, *sitABCD*, *tet*(A), *sul3*	*gyrA* (S83L)	*iroC, iroE, iroN*	IncFIA, IncI2(Delta)
13	S200C2	ST162	*bla*_CTX-M-15_, *bla*_TEM-1B_, *mcr-1.1*, *aac(3)-IIa*, *aadA2*, *aadA24*, *aph(3″)-Ib*, *aph(3′)-Ia*, *aph(6)-Id*, *dfrA14*, *cmlA1*, *erm(B), mph(A)*, *sitABCD*, *tet(A)*, *sul3*	*gyrA* (D87N), *gyrA* (S83L), *parC* (S80I)	*yagW/ecpD, yagX/ecpC, yagZ/ecpA, ykgK/ecpR*	IncFIB(AP001918), IncI2(Delta), IncY
14	S261C1	ST195	*bla*_CTX-M-55_, *bla*_TEM-176_, *mcr-1.1*, *aph(3″)-Ib*, *aph(3′)-Ia*, *aph(6)-Id*, *aadA1*, *aadA2*, *cmlA1*, *fosA3*, *qnrS13*, *sul3*	*gyrA* (D87N), *gyrA* (S83L), *parC* (S80I)	*fimE, fimI, yagZ/ecpA*	ColE10, IncI(Gamma), IncX1, IncX4, p0111
15	S296C	ST10	*bla*_TEM-176_, *ant(3″)-Ia*, *aph(3′)-Ia*, *mcr-1.1*, *fosA4*, *qnrS1*, *sul3*	*gyrA* (D87N), *gyrA* (S83L), *parC* (S80I)	*yagW/ecpD, yagZ/ecpA*	IncI1-I(Alpha), IncI2, IncX1, IncX4, p0111

In addition, we investigated mutations in the *parC*, *gyrA*, and *parE* genes, which are known to contribute to quinolone resistance (Table 2). Remarkably, 13 of the 15 *mcr-1*-positive *E. coli* isolates harbored at least one mutation within these genes. The most frequently observed mutations were *parC* (S80I) and *gyrA* (S83L), identified in 10 and 9 isolates, respectively. Additional mutations included *gyrA* (D87N) in six isolates; *gyrA* (D87Y) and *parC* (S80R) in two isolates each; and *parC* (E84G) and *parE* (S458A), each detected in a single isolate.

### 3.3 Virulence profile of *mcr-1.1* producing *E. coli* isolates

Analysis of the virulence gene profiles among the isolates revealed significant diversity in the presence and distribution of key virulence factors (Table 2). Adhesion-related genes, particularly *fimE*, *fimI*, and the *ecp* gene cluster (*yagW/ecpD*, *yagZ/ecpA*, and *ykgK/ecpR*), were the most commonly detected across isolates, suggesting that bacterial attachment mechanisms are widespread among the studied strains. Several isolates, notably S48C and S167C1, exhibited a high burden of iron acquisition systems, including the *iro* operon (*iroC*, *iroE*, *iroN*) and the *ybt* locus (*ybtA*, *ybtE*, *ybtP*, *ybtQ*, *ybtT*, *ybtU*, *ybtX*), along with siderophore-associated genes (*fyuA*, *irp1*, *irp2*), Toxin-associated genes, such as *astA*, *vat*, and *pic*, were identified in isolates like S99C, S131C, and S136C2.

### 3.4 Plasmid characterization and genetic context of *mcr-1.1* gene

All *mcr-1.1*-producing *E. coli* isolates were found to carry at least one plasmid replicon (Table 2). Among the detected replicon types, IncF and IncI plasmids were the most prevalent. The IncFIB(AP001918) replicon was identified in nine isolates, followed by IncFII(pHN7A8) and IncFII, each present in three isolates. IncFIA and IncFIB(pLF82-PhagePlasmid) were detected in two and one isolate, respectively. IncI plasmids were the second most frequent group, with IncI2(Delta) identified in 10 isolates, IncI1-I(Alpha) in five, IncI(Gamma) in two, and IncI2 in one isolate. Other plasmid types included p0111 in six isolates, IncX1 in four, and both IncY and IncX4 in three isolates each. Additionally, IncHI2, ColpVC, and IncB/O/K/Z replicons were found in two isolates each, while IncHI2A, Col(BS512), and ColE10 were detected in one isolate each.

**Figure 1:**
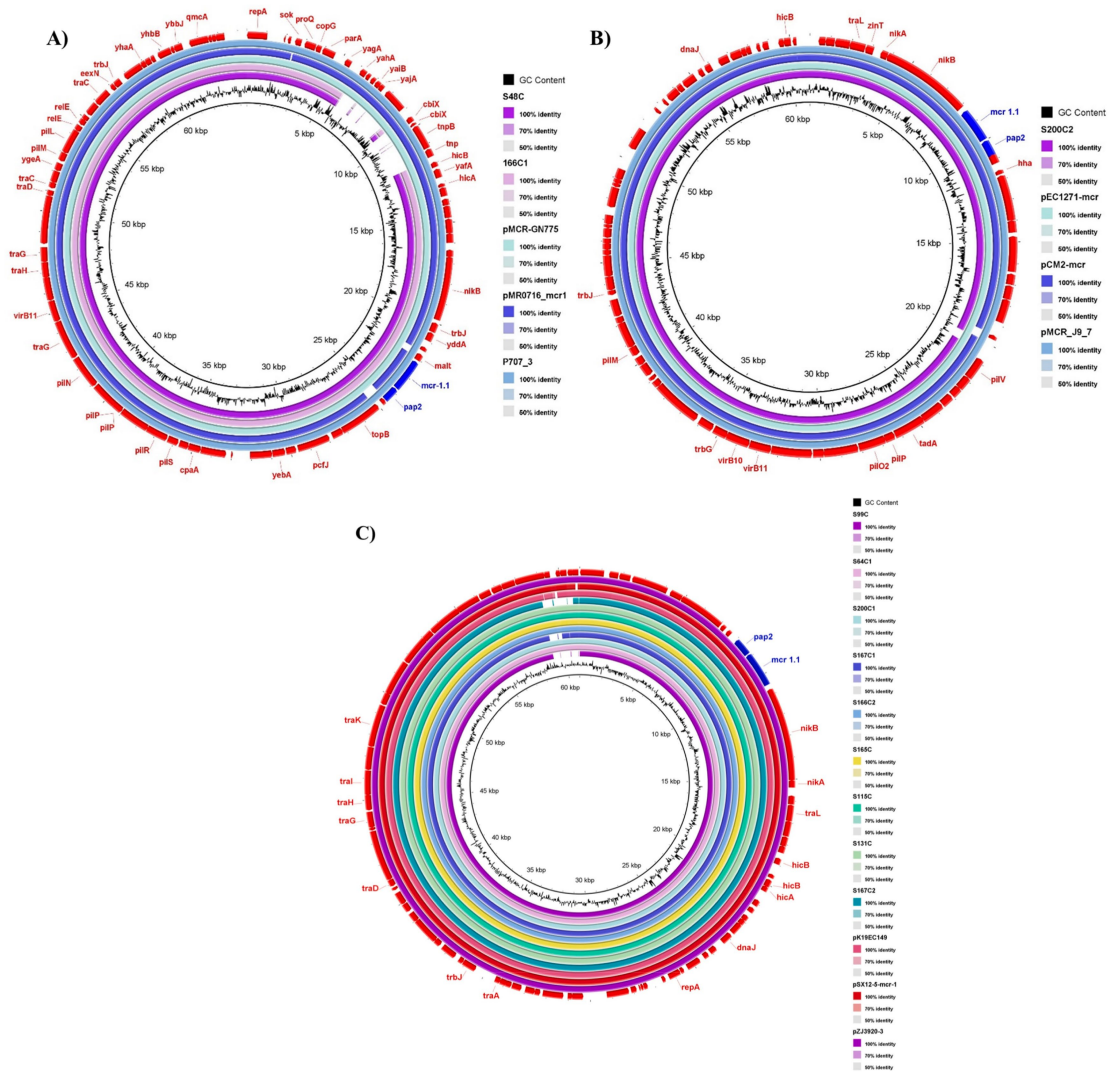
Comparative analysis of 12 *mcr-1.1* IncI2 plasmids carrying this study and 9 plasmids with high similarity in the GenBank database. **(A)** IncI2 plasmids from *Escherichia coli* isolates S48C and S166C1 showed high identity (99.7% % identity; 94–96% coverage) to plasmid p707_3 (GenBank: OY856302.1), pMCR-GN775 (GenBank: KY471307.1), and pMR0716_mcr1 (GenBank: CP018106.1) from humans. **(B)** IncI2 plasmid from *E. coli* isolate S200C2 showed high identity (99–100% identity; 99.99–100% coverage) to plasmids pMCR_J9_7 (GenBank: CP075067.1), pEC1271-mcr (GenBank: OM839890.1), and pCM2-mcr (GenBank: CP158310.1) from swine, human, and chicken sources, respectively. **(C)** IncI2 plasmids from *E. coli* isolates S99C, S64C1, S167C1, S200C1, S166C2, S165, S115C, S131C, and S167C2 showed nearly identical sequences (100% identity; 99.94–99.98% coverage) to plasmids pZJ3920-3 (GenBank: CP020548.1), pSX12-5-mcr-1 (GenBank: CP142786.1), and pK19EC149 (GenBank: CP050290.1) from human, sheep, and dog sources, respectively.

**Figure 2:**
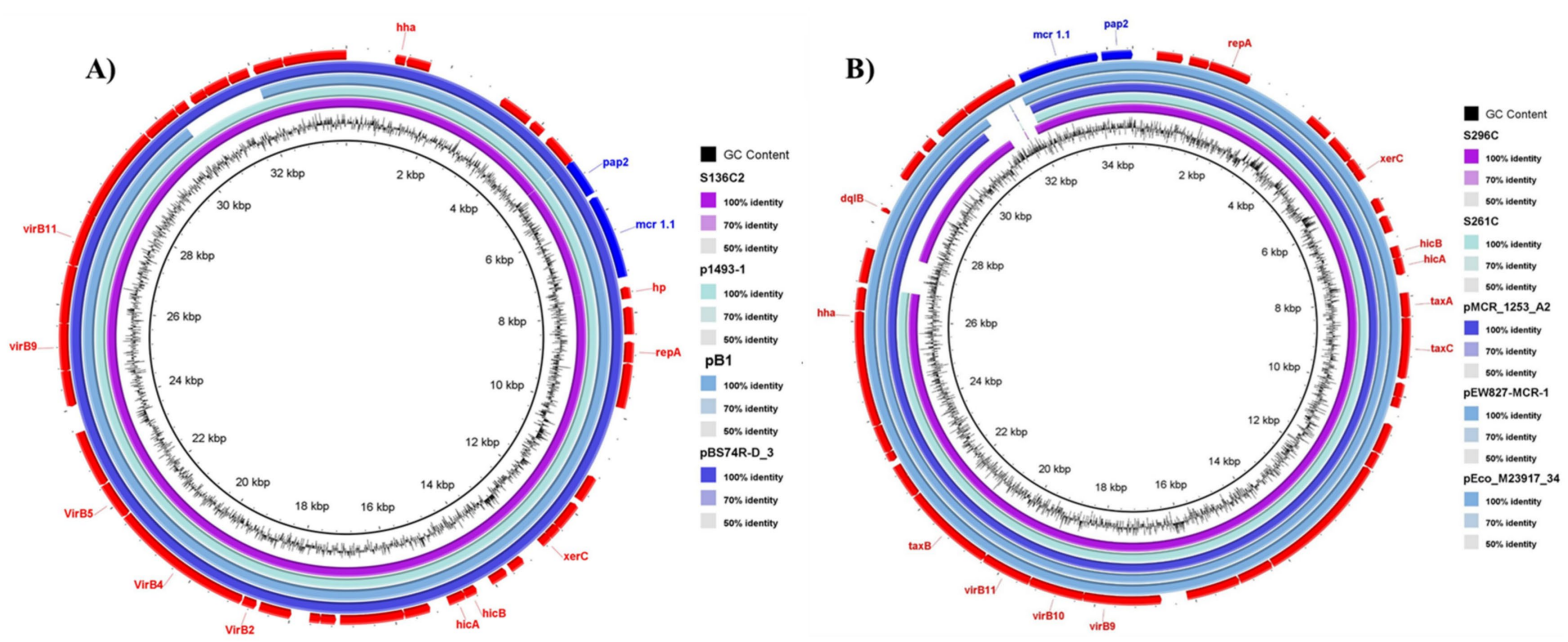
Comparative analysis of 3 *mcr-1.1* IncX4 plasmids carrying this study and 6 plasmids with high similarity in the GenBank database. **(A)** IncX4 plasmid from *E. coli* isolate S136C2 showed high sequence similarity (99.7% identity; 99.7% coverage) to plasmids pBS74R-D_3 (GenBank: CP063335.1), p1493-1 (GenBank: CP019072.1), and pB1 (GenBank: LC479452.1) from human and environmental sources in Switzerland, China, and Japan, respectively. **(B)** IncX4 plasmids from *E. coli* isolates S296C and S261C showed 100% identity and 95–97% coverage with plasmids pEco_M23917_34 (CP133915.1), pEW827-MCR-1 (MW836072.1), and pMCR_1253_A2 (MT929278.1) from human, environmental, and food sources in Argentina, Brazil, and Germany, respectively.

In this study, all *mcr-1.1*-producing *E. coli* isolates were found to carry at least one plasmid replicon (Table 2). In all cases, the IncI2 and IncX4 plasmids carrying *mcr-1.1* were inferred from contigs generated by short-read assembly, with connectivity supported by read-pair links during the Unicycler assembly process. Comparative analysis with publicly available plasmid sequences revealed high similarity to a diverse range of *E. coli* plasmids originating from various sources across multiple countries (Figures 1, 2). Notably, the IncI2 plasmids from *E. coli* isolates S48C and S166C1 exhibited 99.7% sequence identity and 96% coverage with the IncI2 plasmid p707_3 from *E. coli* strain EC7072022BGR, isolated from human urine in Bulgaria in 2022 (GenBank accession: OY856302.1). These plasmids also closely matched plasmid pMCR-GN775 from a clinical *E. coli* isolate in Canada (99.7% identity, 96% coverage; KY471307.1) and plasmid pMR0716_mcr1 from *E. coli* strain MRSN352231, recovered from a clinical sample in Germany in 2016 (99.7% identity, 94% coverage; CP018106.1) (Figure 1A). Additionally, the IncI2 plasmid from isolate S200C2 showed 99–100% identity and nearly complete coverage (99.99–100%) with several plasmids, including pMCR_J9_7 from *E. coli* EC_J_9 recovered from swine intestine in China in 2016 (CP075067.1), pEC1271-mcr from *E. coli* EC1271 isolated from a child’s feces in Taiwan in 2017 (OM839890.1), and pCM2-mcr from *E. coli* CM2 recovered from retail chicken meat in China in 2019 (CP158310.1) (Figure 1B). Furthermore, the IncI2 plasmids from isolates S99C, S64C1, S167C1, S200C1, S166C2, S165, S115C, S131C, and S167C2 exhibited nearly identical sequences (100% identity; 99.94–99.98% coverage) to plasmid pZJ3920-3 from *E. coli* strain ZJ3920 recovered from human bile in China in 2015 (CP020548.1), pSX12-5-mcr-1 from a sheep anal swab in China in 2020 (CP142786.1), and pK19EC149 from feces of a diarrheic dog in South Korea in 2019 (CP050290.1) (Figure 1C).

With respect to *mcr-1.1* genes located on IncX4 plasmids, *E. coli* isolate S136C2 exhibited high sequence similarity (99.7% identity and 99.7% coverage) to several previously reported IncX4 plasmids. These include plasmid pBS74R-D_3 from *E. coli* strain BS74R-D, recovered from a human rectal swab in Switzerland in 2018 (GenBank accession: CP063335.1); plasmid p1493-1 from *E. coli* strain CRE1493, isolated from a human rectal swab in China in 2013 (CP019072.1); and plasmid pB1 from *E. coli* B1, recovered from municipal wastewater influent in Japan in 2018 (LC479452.1) (Figure 2A). Moreover, IncX4 plasmids from isolates S296C and S261C displayed 100% identity with 95–97% coverage to plasmid pEco_M23917_34 from *E. coli* strain M23917, recovered from a human urine sample in Argentina in 2018 (CP133915.1); plasmid pEW827-MCR-1 from *E. coli* strain EW827, isolated from an urban stream in Brazil in 2020 (MW836072.1); and plasmid pMCR_1253_A2 from *E. coli* strain 1253_17_A2, recovered from retail raw turkey meat imported from Germany into the Czech Republic (MT929278.1) (Figure 2B).

**Figure 3:**
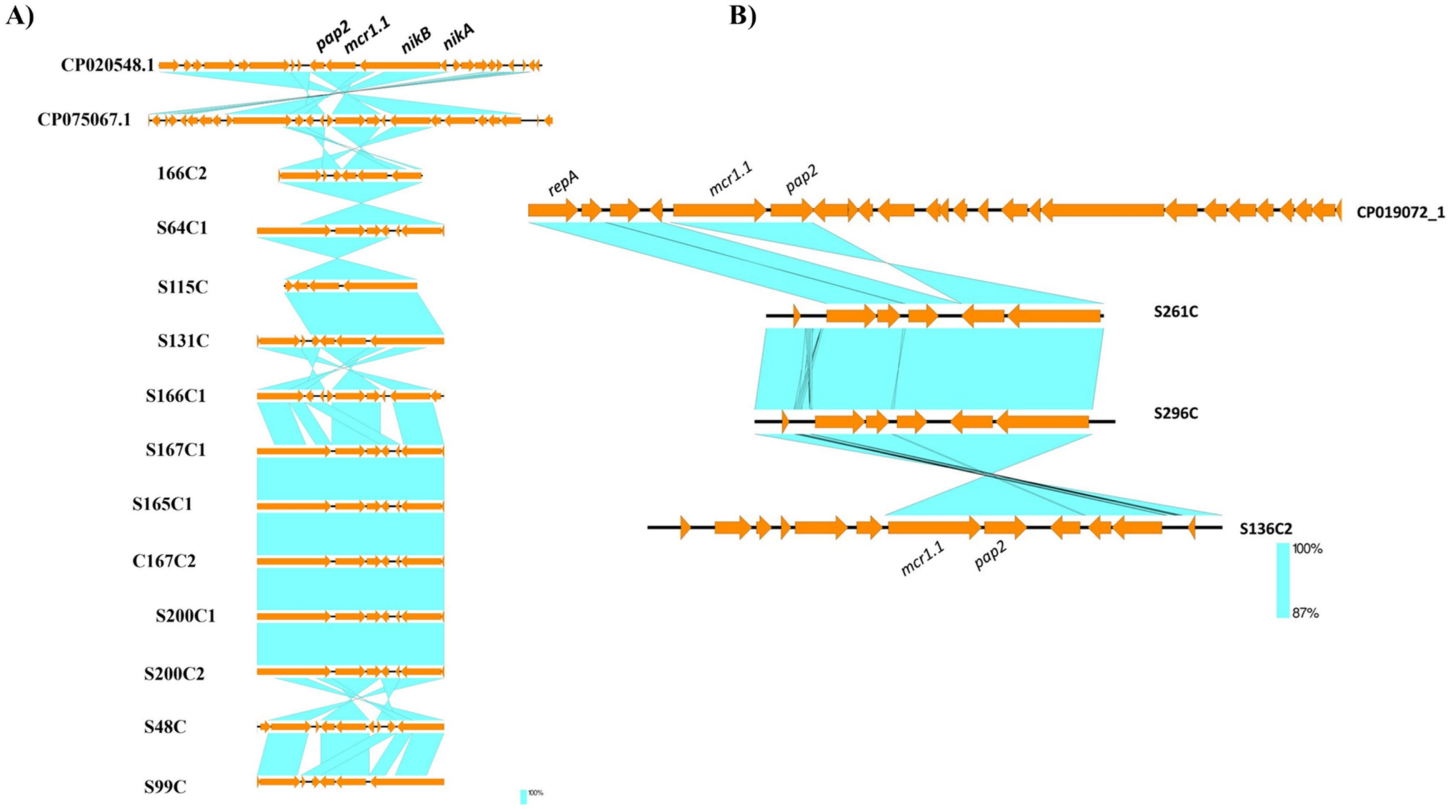
Schematic representation of the genetic environments of surrounding *mcr-1.1* identified from the draft genome sequences of *Escherichia coli* isolated in this study. **(A)** represents the genetic environments of surrounding *mcr-1.1* identified on the IncI2 plasmids. **(B)** represents the genetic environments of surrounding *mcr-1.1* identified on the IncX4 plasmids. The genetic environment surrounding *mcr-1.1* genes was visualized using Easyfig.

Genetic mapping of the *mcr-1.1* gene, as illustrated in Figures 1–3, revealed distinct genetic contexts associated with different plasmid types. In IncI2 plasmids, the *mcr-1.1* gene was consistently flanked upstream by *nikA* (encoding a plasmid mobilization relaxosome protein) and *nikB* (encoding a relaxase), while the *pap2* gene was located downstream (Figures 1, 3). Similarly, in IncX4 plasmids, the *pap2* gene was identified downstream of *mcr-1.1* in the isolates (Figures 2, 3).

### 3.5 Clonality of the isolates

**Figure 4:**
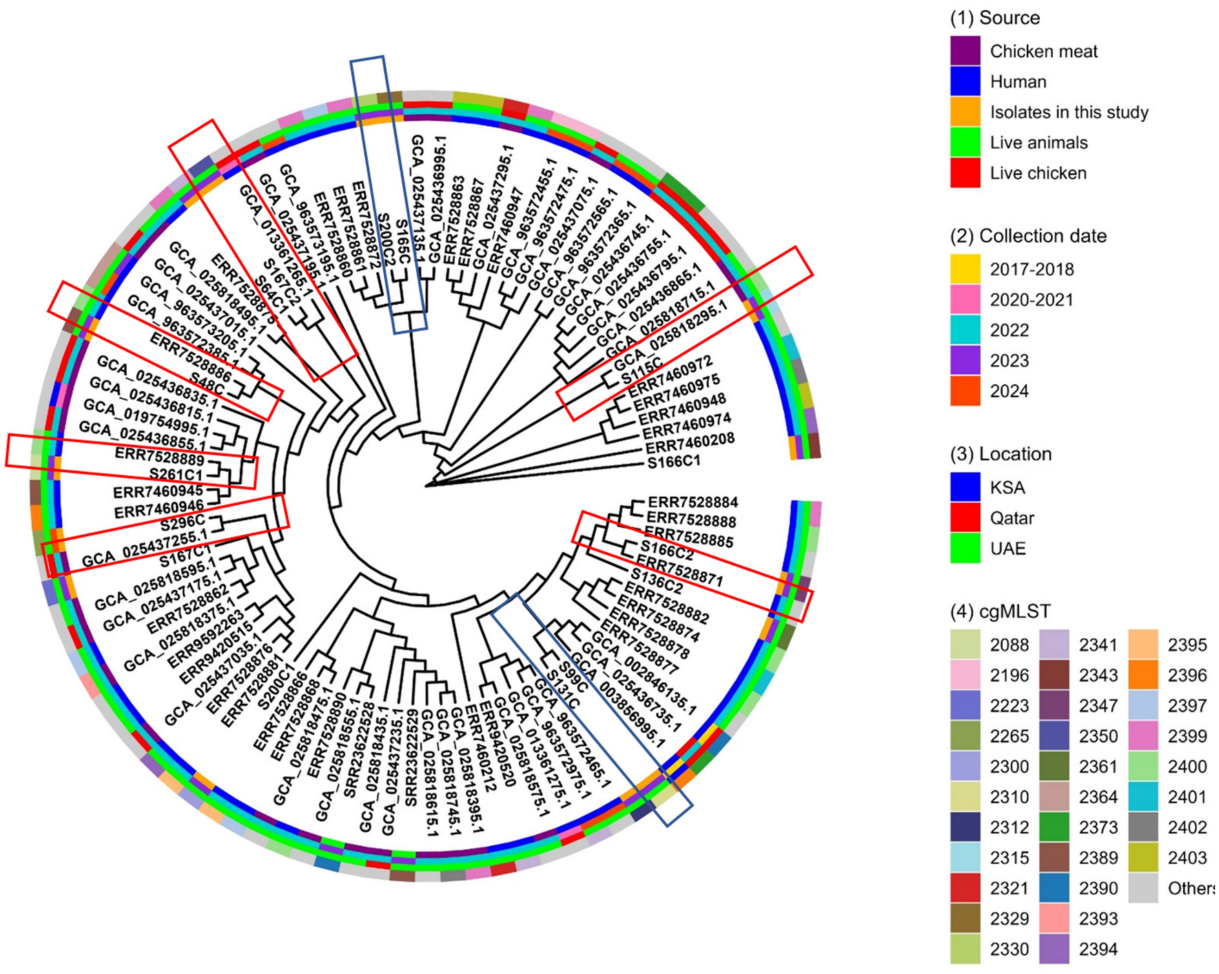
Core genome multilocus sequence typing (cgMLST) analysis of *mcr-1.1*-carrying multidrug-resistant *Escherichia coli* isolates identified in this study, alongside 80 publicly available *mcr-1.1*-positive *E. coli* genomes retrieved from the NCBI database. These comparative genomes were recovered from the United Arab Emirates (*n* = 57), Qatar (*n* = 21), and Saudi Arabia (*n* = 2). Isolates from this study that are closely related to each other are highlighted with blue squares, while those showing genetic relatedness to external isolates are marked with red squares.

Whole-genome sequencing analysis revealed that the 15 *E. coli* isolates belonged to 12 previously characterized STs. The most prevalent sequence types were ST10, ST117, and ST162, each detected in 2 of the 15 isolates (13.3%). The remaining isolates were assigned to ST189, ST46, ST359, ST457, ST101, ST354, ST2172, ST1771, and ST195, each represented by a single isolate. Core genome multilocus sequence typing (cgMLST) demonstrated that the colistin-resistant *E. coli* isolates S99C/S131C (ST117) and S165C/S200C2 (ST162) shared identical genotypes (Figure 4). Several isolates exhibited close genetic relatedness to previously reported *E. coli* strains from the UAE and neighboring countries, originating from clinical and food sources. This clustering in the Minimum Spanning Tree (Figure 4) suggests potential regional dissemination of these specific *mcr-1.1-*carrying clones across the UAE and neighbouring countries. Specifically, isolate S115C (ST359) was closely related to strain GCA_025818295.1 (ST359), isolated from retail chicken meat in the UAE in 2022; isolate S166C2 (ST354) clustered with ERR7528871 (ST354), recovered from a human case in the UAE in 2022; and isolate S296C (ST10) showed close similarity to strain GCA_025437255.1 (ST10), isolated from chicken meat in Qatar in 2022 (Figure 4). In addition, isolate S261C1 (ST195) was related to strain ERR7528889 (ST1630) from a human in the UAE in 2022, while isolate S48C (ST189) aligned with ERR7528886 (ST165) from a human in the UAE in the same year. Isolates S64C1 (ST46) and S167C2 (ST2172) also showed close relatedness to strain GCA_013361265.1 (ST540), previously recovered from human cases in Qatar between 2020 and 2021 (Figure 4).

## 4 Discussion

Our finding aligns with global reports indicating that poultry production systems are one of the major ecological niches for the emergence and dissemination of *mcr* genes, likely due to extensive antibiotic use in intensive farming practices (7, 27). The absence of *mcr*-positive isolates in human or other animal sources in this study may reflect either a limited spill over or under-detection, but it highlights the need for enhanced One Health surveillance across interconnected reservoirs. Phenotypically, the confirmed MDR -based on their non-susceptibly to at least one antimicrobial in three or more antimicrobial categories- and ESBL production among all *mcr*-carrying isolates is particularly concerning, as it suggests co-selection and co-resistance mechanisms driven by mobile genetic elements (28). Notably, all isolates remained susceptible to carbapenems, which is consistent with previous studies indicating that *mcr*-positive *E. coli* can retain susceptibility to last-line *β*-lactams unless co-harboring carbapenemase genes (29). Additionally, the high resistance rates to cephalosporins (CRO, CFP, and FOX), tetracycline, and quinolones further underscore the broad resistance profiles of these isolates and reflect patterns often associated with IncI2 or IncX4 plasmids co-carrying *mcr-1* and other resistance determinants (28, 29). These phenotypic profiles highlight the complexity of treating infections caused by such strains and reinforce the urgent need for antimicrobial stewardship and genomic surveillance in poultry systems to mitigate the spread of *mcr*-mediated resistance.

Whole-genome sequencing of the *mcr-1.1*-positive *E. coli* isolates revealed a complex and alarming resistome, characterized by the co-occurrence of multiple ESBL genes and a diverse array of additional AMR determinants. The high prevalence of *bla*_CTX-M_ genes, particularly *bla*_CTX-M-55_ and *bla*_CTX-M-15_, underscores the dominance of these variants in poultry-associated *E. coli*, which is consistent with global reports linking *bla*_CTX-M_*-*type enzymes with plasmid-mediated dissemination in food animals (29, 30). The concurrent presence of multiple *bla*_TEM_ alleles and *bla*_SHV-12_ further reflects the historical accumulation of *β*-lactamase genes, potentially increasing the resistance spectrum and complicating phenotypic detection. Beyond β-lactams, the isolates harbored resistance determinants against nearly all major antibiotic classes, including aminoglycosides (*aph(3′)-Ia*, *aadA2*), quinolones (*qnrS1*), sulfonamides (*sul3*), tetracyclines (*tet(A)*), and phenicols (*cmlA1*, *floR*), indicating strong selective pressures in poultry production environments and facilitating the persistence of MDR clones (6–8).

Importantly, mutations in chromosomal quinolone resistance-determining regions (QRDRs), such as *parC* (S80I) and *gyrA* (S83L, D87N), were widespread among the isolates, consistent with stepwise accumulation of mutations that confer high-level fluoroquinolone resistance (19, 20). These findings highlight the dual role of plasmid-mediated resistance genes and chromosomal mutations in driving fluoroquinolone resistance, which is particularly concerning given the role of fluoroquinolones in human medicine. The detection of disinfectant resistance genes (*sitABCD*) in the majority of isolates may further promote co-selection of AMR traits in environments exposed to biocides, a phenomenon increasingly recognized in agricultural and clinical settings (31). Together, the extensive co-resistance observed in these *mcr-1.1*-carrying isolates emphasizes their potential to act as high-risk clones capable of surviving multiple therapeutic and environmental pressures.

The virulence profiling of *mcr-1.1*-producing *E. coli* isolates revealed key traits related to adhesion, iron acquisition, and toxin production. The widespread presence of adhesion genes (*fimE*, *fimI*, *ecp* cluster) suggests an important role in chickens epithelial colonization, which supports persistence and invasion (32). Additionally, isolates such as S48C and S167C1 carried numerous iron acquisition genes (*iro* operon, *ybt* locus, *fyuA*, *irp1*, *irp2*), which are crucial for survival in iron-limited environments and associated with extraintestinal pathogenic *E. coli* (ExPEC) strains (33). Furthermore, the presence of toxin genes (*astA*, *vat*, *pic*) in some isolates suggests potential for tissue damage, immune evasion, and diarrheal illness (32, 33). This highlights the pathogenic versatility of *mcr-1.1*-positive *E. coli* and underscores the importance of ongoing surveillance to monitor their clinical and zoonotic risks.

Plasmid characterization in this study reveals a wide diversity of plasmid replicons, with IncF and IncI plasmids being the most prevalent among the *mcr-1.1*-producing isolates. These findings are consistent with previous studies showing that *mcr-1* is often carried by plasmids, which are mobile genetic elements facilitating horizontal gene transfer (6–8, 11) The IncF plasmids detected in this study, particularly IncFIB(AP001918), IncFII(pHN7A8), and IncFII, have been widely reported in clinical *E. coli* isolates (34). IncF plasmids are known to harbor a broad range of AMR genes, including those encoding for ESBLs, carbapenemases, and other resistant determinants. Their high prevalence among *mcr-1.1*-producing isolates is concerning, as these plasmids are capable of mobilizing resistance genes across bacterial populations and environments.

IncI plasmids, especially the IncI2 type, were also frequently associated with *mcr-1.1*-bearing isolates. These plasmids are recognized for their high mobility and their association with the dissemination of AMR genes (29, 35). In this study, the high sequence identity of IncI2 plasmids from *E. coli* isolates to plasmids isolated from a diverse range of sources, including human, swine, and retail chicken meat in China, as well as from human bile in China and South Korea, further underscores the widespread distribution of *mcr-1.1*-bearing IncI2 plasmids. These findings are consistent with previous reports indicating that the IncI2 plasmid is associated with the colistin resistance gene *mcr-1* and its variants *mcr-1.3* and *mcr-1.5*. Moreover, this association has been documented in both human and animal sources across China, Japan, Denmark, Spain, and the UAE (36, 37). The close sequence identity between the plasmids from this study and those isolated from human, animal, and environmental sources (such as those from Bulgaria, Canada, and Germany) suggests that these plasmids, and by extension, *mcr-1.1*, have disseminated globally (7).

The detection of *mcr-1.1* on IncX4 plasmids is also noteworthy. IncX4 plasmids, although less commonly studied than IncF and IncI plasmids, have been identified as important vectors for the dissemination of AMR genes (36). The high sequence similarity between IncX4 plasmids from isolates in this study and those from clinical and environmental sources across the globe (Argentina [GenBank accession number CP133915.1], Brazil [GenBank accession number MW836072.1], and Japan [GenBank accession number LC479452.1]) further supports the hypothesis of a widespread environmental and clinical dissemination of *mcr-1.1*.

Genetic mapping of the *mcr-1.1* gene in this study revealed distinct genetic contexts depending on the plasmid type. In IncI2 plasmids, the *mcr-1.1* gene was consistently flanked by mobilization genes *nikA* and *nikB*, which encode for plasmid mobilization and relaxation, respectively. These genes are essential for the efficient transfer of plasmids between bacteria, promoting the horizontal spread of the *mcr-1.1* gene (38). Additionally, the downstream presence of the *pap2* gene suggests that the genetic context of *mcr-1.1* on IncI2 plasmids may facilitate its stable inheritance and mobilization (10). Furthermore, previous studies have shown that *mcr-1*-carrying IncX4-type plasmids exhibit a highly conserved structure, typically comprising the *mcr-1–pap2* gene cassette, type IV secretion system (T4SS) components, plasmid replication and maintenance genes, toxin-antitoxin (TA) modules, and the insertion sequence *IS26* (39).

The genomic characterization of the 15 *mcr-1.1*-positive *E. coli* isolates revealed high clonal diversity, with assignment to 12 distinct STs, indicative of multiple independent introductions rather than expansion from a single source. Notably, the identification of high-risk clones—ST10, ST117, and ST162—each in two isolates, reflects their epidemiological significance as globally distributed lineages involved in zoonotic transmission and antimicrobial resistance dissemination (40). These lineages are commonly recovered from poultry, humans, and food products, suggesting their ability to act as reservoirs and mediators of *mcr-1*-driven resistance within the One Health continuum (40). The detection of identical cgMLST profiles among isolate pairs S99C/S131C (ST117) and S165C/S200C2 (ST162) provides compelling evidence of recent clonal spread, possibly originating from shared sources or direct transmission along the poultry-human-food interface. ST117, a well-documented avian pathogenic *E. coli* lineage, is frequently associated with broiler chickens and retail meat and has demonstrated the capacity to colonize and infect humans (40). Similarly, ST162 has been widely reported in food-producing animals and human infections, often co-carrying *mcr-1* and ESBL genes. These findings suggest that colistin-resistant *E. coli* clones are not only circulating within poultry environments but are also capable of entering and persisting in the human population, likely via the food chain.

More strikingly, our data reveal strong genomic relatedness between isolates from chicken cloacal samples and previously reported strains from human and retail meat sources in the UAE and Qatar. Isolate S115C (ST359) was nearly identical to strain GCA_025818295.1, previously recovered from chicken meat in the UAE in 2022, indicating the persistence and potential expansion of this clone in the local food supply chain. Isolate S166C2 (ST354), closely matching a human-derived strain (ERR7528871) from the UAE, highlights possible direct or indirect transmission from poultry to humans, perhaps mediated by improper meat handling or consumption of contaminated products. Notably, isolate S296C (ST10) was genetically related to a chicken meat isolate from Qatar (GCA_025437255.1), highlighting the role of ST10 as a globally disseminated pandemic clone associated with the spread of *mcr*-harboring *E. coli* across diverse hosts and environments. The detection of isolate S166C1 (ST101), genetically identical to a human-associated strain (ERR7460208) from the UAE, further emphasizes the role of high-risk clones with epidemic potential. ST101 is a globally emerging lineage, often co-harboring *mcr*, ESBLs, and carbapenemases, and has been implicated in intestinal and extraintestinal infection (41). Its presence in poultry environments signals a worrisome trajectory for the convergence of resistance and virulence traits across sectors.

Importantly, the identification of isolates with high genetic similarity across national borders (UAE and Qatar), including ST10 and ST46-related strains, suggests that the observed transboundary spread of *mcr-1.1*-positive *E. coli* clones may be attributed to the similar structure and operational practices of the poultry sectors in both countries. Given their comparable climates, both the UAE and Qatar rely heavily on importing one-day-old chicks and other key inputs along the poultry value chain from a limited number of source countries and follow harmonized GCC standards for importation and biosecurity controls. This situation mirrors findings in other parts of the world where international dissemination of *mcr*-bearing strains has been facilitated through the globalized food supply chain and migratory livestock trade (42). Our results highlight the interconnectedness of antimicrobial resistance across sectors and geographic regions, and call for a coordinated regional One Health surveillance and control program that spans veterinary, clinical, and food safety domains.

Although this study focused solely on the detection of the *mcr-1* gene, it is now recognized that at least 10 *mcr* allelic variants (*mcr-1* to *mcr-10*) have been described in different bacterial species and hosts worldwide. Our decision to target *mcr-1* was based on its established predominance and its historical role as the first and most widely disseminated plasmid-mediated colistin resistance determinant. In addition, regional surveillance data and published literature indicate that *mcr-1* remains the principal variant reported from the Middle East and surrounding regions. Nevertheless, we acknowledge that restricting the screening to *mcr-1* may underestimate the overall burden of *mcr*-mediated colistin resistance in our collection. Nevertheless, several limitations should be acknowledged. First, the use of short-read sequencing limited our ability to achieve complete plasmid closure and fully resolve the genetic context of *mcr*-carrying elements. Second, the restricted screening to *mcr-1* may have underestimated the diversity and overall prevalence of *mcr*-mediated colistin resistance. Third, the absence of detailed epidemiological linkage data prevented us from assessing potential transmission pathways between human, animal, and environmental sources. Finally, a potential sampling bias related to the origin and temporal distribution of isolates may have influenced the representativeness of our findings. Future studies incorporating multiplex or whole-genome approaches to detect all known *mcr* variants will be essential to provide a more comprehensive understanding of the resistance landscape.

## 5 Conclusion

This study highlights poultry establishments in the UAE as important reservoirs of *mcr-1.1*-harboring *E. coli*. The detection of *mcr-1.1* in broiler isolates with multidrug resistance and diverse plasmid types (IncI2, IncX4) reflects the potential for horizontal gene transfer and global dissemination. The presence of high-risk clones (ST10, ST117, and ST162) underscores the need for vigilant surveillance across the food–animal–human interface. Genomic relatedness between poultry, human, and food isolates suggests possible cross-sector transmission. These findings reinforce the urgent need for integrated One Health surveillance and antimicrobial stewardship to mitigate the spread of *mcr*-mediated resistance.

## Data Availability

The original contributions presented in the study are publicly available. This data can be found here: GenBank, BioProject ID, PRJNA1221085.

## Footnotes

1
https://github.com/tseemann/abricate

2
https://github.com/tidyverse/dplyr

3
https://github.com/tidyverse/tidyr

4
https://github.com/tidyverse/ggplot2

5
https://github.com/ElbediwiM/cgMLST-tree
