# Supplementary material for: Molecular characterization of mcr-1.1-harboring multidrug-resistant Escherichia coli isolates from chicken in the United Arab Emirates: implications for one health surveillance
Source: Front Vet Sci. 2026 Jan 20;12:1714397. doi: 10.3389/fvets.2025.1714397 (PMC12866622; doi:10.3389/fvets.2025.1714397)
Supplement: Supplementary file 1 [file Data_Sheet_1.docx]

Supplementary Material

**Supplementary Table 1:** Isolates used to evaluate the phylogenomic relationships in this study

| **id** | **Source** | **MLST** | **Location** | **Collection date** | **cgMLST** |
| --- | --- | --- | --- | --- | --- |
| GCA_002846135.1 | Human | 95 | Qatar | 2017-2018 | 2390 |
| GCA_003856995.1 | Human | 131 | KSA | 2017-2018 | 2396 |
| GCA_013361265.1 | Human | 540 | Qatar | 2020-2021 | Others |
| GCA_013361275.1 | Human | 115 | Qatar | 2020-2021 | Others |
| GCA_019754995.1 | Chicken meat | 3270 | KSA | 2020-2021 | Others |
| GCA_025436735.1 | Live chicken | 355 | Qatar | 2022 | 2373 |
| GCA_025436745.1 | Live chicken | 602 | Qatar | 2022 | 2373 |
| GCA_025436755.1 | Live chicken | 602 | Qatar | 2022 | 2373 |
| GCA_025436795.1 | Live chicken | 602 | Qatar | 2022 | Others |
| GCA_025436815.1 | Chicken meat | 48 | Qatar | 2022 | Others |
| GCA_025436835.1 | Chicken meat | 48 | Qatar | 2022 | Others |
| GCA_025436855.1 | Chicken meat | 3270 | Qatar | 2022 | Others |
| GCA_025436865.1 | Live chicken | 602 | Qatar | 2022 | Others |
| GCA_025436995.1 | Chicken meat | 295 | Qatar | 2022 | Others |
| GCA_025437015.1 | Chicken meat | NA | Qatar | 2022 | Others |
| GCA_025437035.1 | Chicken meat | 744 | Qatar | 2022 | Others |
| GCA_025437075.1 | Chicken meat | 155 | Qatar | 2022 | Others |
| GCA_025437135.1 | Chicken meat | 295 | Qatar | 2022 | Others |
| GCA_025437175.1 | Chicken meat | NA | Qatar | 2022 | Others |
| GCA_025437195.1 | Chicken meat | 6448 | Qatar | 2022 | Others |
| GCA_025437235.1 | Chicken meat | 1011 | Qatar | 2022 | Others |
| GCA_025437255.1 | Chicken meat | 10 | Qatar | 2022 | Others |
| GCA_025437295.1 | Chicken meat | 224 | Qatar | 2022 | 2321 |
| ERR7460208 | Human | 101 | UAE | 2022 | 2394 |
| ERR7460212 | Human | 4633 | UAE | 2022 | 2321 |
| ERR7460945 | Human | 48 | UAE | 2022 | 2389 |
| ERR7460946 | Human | 48 | UAE | 2022 | 2396 |
| ERR7460947 | Human | 533 | UAE | 2022 | 2399 |
| ERR7460948 | Human | 101 | UAE | 2022 | 2402 |
| ERR7460972 | Human | 101 | UAE | 2022 | Others |
| ERR7460974 | Human | 101 | UAE | 2022 | 2403 |
| ERR7460975 | Human | 101 | UAE | 2022 | 2401 |
| ERR7528860 | Human | 12220 | UAE | 2022 | 2399 |
| ERR7528861 | Human | 12220 | UAE | 2022 | 2397 |
| ERR7528862 | Human | 1290 | UAE | 2022 | 2397 |
| ERR7528863 | Human | 1196 | UAE | 2022 | 2403 |
| ERR7528866 | Human | 1140 | UAE | 2022 | 2395 |
| ERR7528867 | Human | 1196 | UAE | 2022 | 2403 |
| ERR7528868 | Human | 1140 | UAE | 2022 | 2397 |
| ERR7528871 | Human | 354 | UAE | 2022 | Others |
| ERR7528872 | Human | 162 | UAE | 2022 | 2399 |
| ERR7528874 | Human | 1485 | UAE | 2022 | 2401 |
| ERR7528875 | Human | 93 | UAE | 2022 | 2399 |
| ERR7528876 | Human | 1585 | UAE | 2022 | 2394 |
| ERR7528877 | Human | 1485 | UAE | 2022 | Others |
| ERR7528878 | Human | 1485 | UAE | 2022 | 2400 |
| ERR7528881 | Human | 1585 | UAE | 2022 | 2395 |
| ERR7528882 | Human | 1485 | UAE | 2022 | 2400 |
| ERR7528884 | Human | 354 | UAE | 2022 | 2399 |
| ERR7528885 | Human | 354 | UAE | 2022 | Others |
| ERR7528886 | Human | 165 | UAE | 2022 | 2400 |
| ERR7528888 | Human | 354 | UAE | 2022 | 2400 |
| ERR7528889 | Human | 1630 | UAE | 2022 | 2400 |
| ERR7528890 | Human | 1011 | UAE | 2022 | 2400 |
| GCA_025818745.1 | Chicken meat | 1011 | UAE | 2022 | 2402 |
| GCA_025818715.1 | Chicken meat | 602 | UAE | 2022 | Others |
| GCA_025818395.1 | Chicken meat | 1011 | UAE | 2022 | 2399 |
| GCA_025818615.1 | Chicken meat | 1011 | UAE | 2022 | Others |
| GCA_025818595.1 | Chicken meat | 10 | UAE | 2022 | Others |
| GCA_025818575.1 | Chicken meat | NA | UAE | 2022 | Others |
| GCA_025818555.1 | Chicken meat | 1011 | UAE | 2022 | Others |
| GCA_025818475.1 | Chicken meat | 1140 | UAE | 2022 | Others |
| GCA_025818495.1 | Chicken meat | 93 | UAE | 2022 | Others |
| GCA_025818435.1 | Chicken meat | 1011 | UAE | 2022 | Others |
| GCA_025818375.1 | Chicken meat | 1290 | UAE | 2022 | 2393 |
| GCA_025818295.1 | Chicken meat | 359 | UAE | 2022 | 2400 |
| ERR9420515 | Human | 167 | UAE | 2022 | Others |
| ERR9420520 | Human | 1158 | UAE | 2022 | 2341 |
| ERR9592263 | Human | 167 | UAE | 2022 | Others |
| SRR23622528 | Live animals | 1011 | UAE | 2023 | 2390 |
| SRR23622529 | Live animals | 1011 | UAE | 2023 | 2389 |
| GCA_963573205.1 | Human | 206 | UAE | 2023 | 2364 |
| GCA_963572365.1 | Human | 2144 | UAE | 2024 | Others |
| GCA_963572385.1 | Human | 206 | UAE | 2024 | 2364 |
| GCA_963572455.1 | Human | 155 | UAE | 2024 | 2196 |
| GCA_963572465.1 | Human | 2732 | UAE | 2024 | Others |
| GCA_963572475.1 | Human | 155 | UAE | 2024 | 2196 |
| GCA_963572565.1 | Human | 2144 | UAE | 2024 | Others |
| GCA_963572975.1 | Human | 2732 | UAE | 2024 | 2341 |
| GCA_963573195.1 | Human | 23 | UAE | 2024 | Others |
| S48C | Isolates in this study | 189 | UAE | 2023 | 2389 |
| S64C1 | Isolates in this study | 46 | UAE | 2023 | 2341 |
| S99C | Isolates in this study | 117 | UAE | 2023 | 2310 |
| S115C | Isolates in this study | 359 | UAE | 2023 | 2315 |
| S131C | Isolates in this study | 117 | UAE | 2023 | 2312 |
| S136C2 | Isolates in this study | 457 | UAE | 2023 | 2361 |
| S165C | Isolates in this study | 162 | UAE | 2023 | 2329 |
| S166C1 | Isolates in this study | 101 | UAE | 2023 | 2343 |
| S166C2 | Isolates in this study | 354 | UAE | 2023 | 2347 |
| S167C2 | Isolates in this study | 2172 | UAE | 2023 | 2350 |
| S167C1 | Isolates in this study | 2172 | UAE | 2023 | 2223 |
| S200C1 | Isolates in this study | 1771 | UAE | 2023 | 2300 |
| S200C2 | Isolates in this study | 162 | UAE | 2023 | 2330 |
| S261C1 | Isolates in this study | 195 | UAE | 2023 | 2088 |
| S296C | Isolates in this study | 10 | UAE | 2024 | 2265 |

**Supplementary Table S2**: The full assembly's quality metrics of the sequenced isolates

| Assembly | S166_2 | S166_1 | S165 | S136_2 | S131 | S115 | S99 | S296 | S261 | S200_2 | S200_1 | S167_2 | S64_1 | S48 | S167_1 |
| --- | --- | --- | --- | --- | --- | --- | --- | --- | --- | --- | --- | --- | --- | --- | --- |
| # Contigs (≥ 0 bp) | 244 | 189 | 232 | 210 | 212 | 301 | 230 | 208 | 346 | 235 | 229 | 279 | 266 | 543 | 304 |
| # Contigs (≥ 1000 bp) | 112 | 120 | 92 | 84 | 101 | 149 | 112 | 100 | 156 | 97 | 110 | 165 | 118 | 205 | 138 |
| # Contigs (≥ 5000 bp) | 73 | 84 | 61 | 61 | 69 | 96 | 73 | 68 | 93 | 65 | 74 | 120 | 90 | 113 | 74 |
| # Contigs (≥ 10000 bp) | 53 | 68 | 46 | 54 | 57 | 73 | 63 | 61 | 77 | 51 | 57 | 98 | 75 | 92 | 58 |
| # Contigs (≥ 25000 bp) | 42 | 47 | 34 | 45 | 45 | 49 | 44 | 49 | 54 | 37 | 41 | 63 | 56 | 60 | 48 |
| # Contigs (≥ 50000 bp) | 31 | 38 | 26 | 27 | 33 | 32 | 31 | 36 | 33 | 27 | 28 | 34 | 32 | 34 | 32 |
| Total length (≥ 0 bp) | 5501669 | 5503675 | 5410427 | 5229984 | 5254793 | 5465622 | 5364429 | 5139952 | 5132571 | 5388172 | 5282057 | 5275934 | 4905928 | 5790784 | 5217659 |
| Total length (≥ 1000 bp) | 5459138 | 5477941 | 5366567 | 5192430 | 5215620 | 5411491 | 5321921 | 5104716 | 5070406 | 5344494 | 5241105 | 5238797 | 4861202 | 5680592 | 5163221 |
| Total length (≥ 5000 bp) | 5367472 | 5397313 | 5294450 | 5141628 | 5138105 | 5294367 | 5226801 | 5022324 | 4918855 | 5272307 | 5160774 | 5114300 | 4791342 | 5447615 | 5002545 |
| Total length (≥ 10000 bp) | 5224714 | 5279125 | 5192047 | 5086707 | 5043129 | 5127585 | 5154214 | 4970179 | 4809730 | 5175012 | 5045298 | 4955909 | 4675494 | 5299073 | 4892759 |
| Total length (≥ 25000 bp) | 5040445 | 4968852 | 4998524 | 4949590 | 4868120 | 4727054 | 4865615 | 4767808 | 4438145 | 4952894 | 4799202 | 4421750 | 4360897 | 4823962 | 4725290 |
| Total length (≥ 50000 bp) | 4692097 | 4647539 | 4712249 | 4309150 | 4431808 | 4126116 | 4397878 | 4299363 | 3745526 | 4589211 | 4395723 | 3425010 | 3512846 | 3868735 | 4172626 |
| # Contigs | 129 | 140 | 116 | 104 | 121 | 179 | 134 | 121 | 190 | 119 | 136 | 190 | 138 | 259 | 172 |
| Largest contig | 388814 | 270168 | 615853 | 491063 | 451012 | 268971 | 456319 | 313472 | 277665 | 380634 | 534548 | 245158 | 327522 | 330480 | 398310 |
| Total length | 5470527 | 5491505 | 5384044 | 5205799 | 5229493 | 5434057 | 5337026 | 5118164 | 5095796 | 5360734 | 5258962 | 5255969 | 4874292 | 5716728 | 5186704 |
| GC (%) | 50.44 | 50.06 | 50.40 | 50.35 | 50.62 | 50.40 | 50.64 | 50.29 | 50.57 | 50.40 | 50.59 | 50.51 | 50.68 | 50.14 | 50.59 |
| N50 | 167117 | 109156 | 270196 | 156940 | 128255 | 125739 | 128114 | 119974 | 98044 | 193786 | 144018 | 71939 | 90638 | 85860 | 122857 |
| N90 | 28161 | 29424 | 35170 | 35148 | 34342 | 19475 | 29654 | 34883 | 18940 | 31789 | 25452 | 14478 | 22570 | 13259 | 26538 |
| auN | 180343.5 | 133725.9 | 246682.7 | 202475.1 | 165616.7 | 126168.1 | 170655.9 | 132621.1 | 110832.5 | 204519.0 | 228620.2 | 89838.9 | 108741.9 | 112507.4 | 148968.7 |
| L50 | 11 | 15 | 8 | 10 | 12 | 15 | 12 | 14 | 17 | 9 | 9 | 22 | 18 | 19 | 13 |
| L90 | 38 | 46 | 30 | 37 | 40 | 57 | 42 | 44 | 61 | 33 | 39 | 80 | 58 | 79 | 46 |
| # N's per 100 kbp | 0.00 | 0.00 | 0.00 | 0.00 | 0.00 | 0.00 | 0.00 | 0.00 | 0.00 | 0.00 | 0.00 | 0.00 | 0.00 | 0.00 | 0.00 |

**Supplementary Table S3**: Number of antimicrobial-resistant genes *in mcr.1*-producing *E. coli* tested in this study based on whole genome sequencing

| **Antibiotic group** | **Resistance genes** | **Number** |
| --- | --- | --- |
| **β-lactams** | *bla*_CTX-M_ | 13 |
|  | *bla*_TEM_ | 12 |
|  | *bla*_SHV_ | 1 |
| **Aminoglycosides** | *aph(3'')-Ib* | 10 |
|  | *aadA2* | 10 |
|  | *aph(6)-Id* | 10 |
|  | *aph(3')-Ia* | 10 |
|  | *aadA1* | 7 |
|  | *aac(3)-Iid* | 5 |
|  | *aac(3)-Iia* | 4 |
|  | *aadA24* | 3 |
|  | *ant(3'')-Ia* | 3 |
|  | *aadA22* | 2 |
|  | *aadA17* | 1 |
|  | *aac(3)-IV* | 1 |
|  | *aph(4)-Ia* | 1 |
| **Quinolone** | *qnrS1* | 6 |
|  | *qnrS13* | 1 |
| **Trimethoprim** | *dfrA14* | 5 |
|  | *dfrA12* | 2 |
|  | *dfrA1* | 1 |
|  | *dfrA17* | 1 |
| **Sulfonamides** | *sul3* | 12 |
|  | *sul2* | 7 |
|  | *sul1* | 1 |
| **Tetracycline** | *tet(A)* | 13 |
|  | *tet(M)* | 1 |
| **Phenicol** | *cmlA1* | 11 |
|  | *floR* | 9 |
|  | *catA1* | 3 |
| **Macrolide resistance** | *mph(A)* | 4 |
|  | *erm(B)* | 4 |
| **Fosfomycin** | *fosA3* | 4 |
|  | *fosA4* | 1 |
| **Lincosamides** | *lnu(F)* | 1 |
| **Disinfectant** | *sitABCD* | 9 |
|  | *qacE* | 2 |


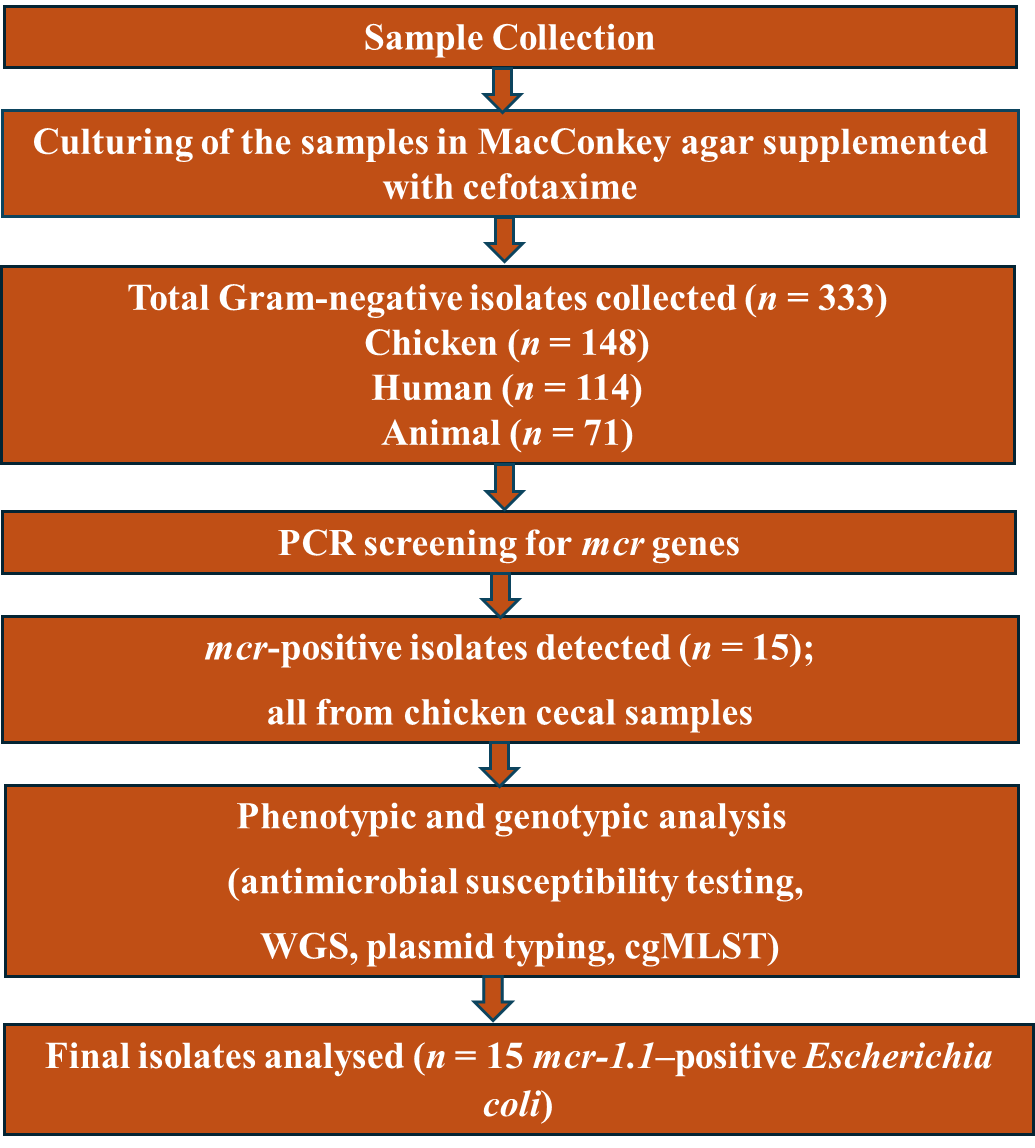


**Supplementary Figure S1**. Workflow of sample collection, screening, and analysis of *mcr-1.1–*positive *Escherichia coli* isolates.
